# Sense and Insensibility – An Appraisal of the Effects of Clinical Anesthetics on Gastropod and Cephalopod Molluscs as a Step to Improved Welfare of Cephalopods

**DOI:** 10.3389/fphys.2018.01147

**Published:** 2018-08-24

**Authors:** William Winlow, Gianluca Polese, Hadi-Fathi Moghadam, Ibrahim A. Ahmed, Anna Di Cosmo

**Affiliations:** ^1^Department of Biology, University of Naples Federico II, Naples, Italy; ^2^Institute of Ageing and Chronic Diseases, University of Liverpool, Liverpool, United Kingdom; ^3^NPC Newton, Preston, United Kingdom; ^4^Department of Physiology, Faculty of Medicine, Physiology Research Centre, Ahvaz Jundishapur University of Medical Sciences, Ahvaz, Iran; ^5^Faculty of Medicine, University of Garden City, Khartoum, Sudan

**Keywords:** clinical anesthetics, gastropods, cephalopods, identified neurons, behavior

## Abstract

Recent progress in animal welfare legislation stresses the need to treat cephalopod molluscs, such as *Octopus vulgaris*, humanely, to have regard for their wellbeing and to reduce their pain and suffering resulting from experimental procedures. Thus, appropriate measures for their sedation and analgesia are being introduced. Clinical anesthetics are renowned for their ability to produce unconsciousness in vertebrate species, but their exact mechanisms of action still elude investigators. In vertebrates it can prove difficult to specify the differences of response of particular neuron types given the multiplicity of neurons in the CNS. However, gastropod molluscs such as *Aplysia*, *Lymnaea*, or *Helix*, with their large uniquely identifiable nerve cells, make studies on the cellular, subcellular, network and behavioral actions of anesthetics much more feasible, particularly as identified cells may also be studied in culture, isolated from the rest of the nervous system. To date, the sorts of study outlined above have never been performed on cephalopods in the same way as on gastropods. However, criteria previously applied to gastropods and vertebrates have proved successful in developing a method for humanely anesthetizing Octopus with clinical doses of isoflurane, i.e., changes in respiratory rate, color pattern and withdrawal responses. However, in the long term, further refinements will be needed, including recordings from the CNS of intact animals in the presence of a variety of different anesthetic agents and their adjuvants. Clues as to their likely responsiveness to other appropriate anesthetic agents and muscle relaxants can be gained from background studies on gastropods such as *Lymnaea*, given their evolutionary history.

## Introduction

Under normal circumstances animals are sensitive to their environment and to changes within it, particularly damaging changes which will elicit fight or flight or withdrawal responses. These capabilities are shared among distant related lineages supporting the proposal that the fundamental mechanisms evolved as adaptive responses to noxious stimuli (Walters and Moroz, 2009; De Lisa et al., 2012). The role of anesthetics is to render subjects insensible to surgical interventions which would normally be perceived as damaging noxious stimuli. Clinical anesthetics are renowned for their ability to produce unconsciousness in vertebrate species, but their exact mechanisms of action still elude investigators (Herold and Hemmings, 2012; Herold et al., 2017b), for which reason a number of model systems have been studied over the years e.g., lipid bilayers (Herold et al., 2017a,b) and invertebrate systems (Winlow, 1984; Franks and Lieb, 1988). However, on the basis of strong evidence, it is now assumed that general anesthetics have limited effects on the properties of lipid bilayers (Herold et al., 2017b) and that anesthetics have specific actions on membrane proteins, both at the cell membrane (Lopes et al., 1998) and intracellularly (Harris, 1981; Winlow et al., 1995; Moghadam and Winlow, 2000). Ideally anesthetics should have no side effects, act as muscle relaxants, as analgesics and as amnesics as well as rendering the individual unconscious. In practice this is difficult to achieve with a single molecule and a cocktail of molecules (Donohue et al., 2013), including hypnotics for sedation (Meerts and Absalom, 2013) and muscle relaxants (Khirwadkar and Hunter, 2012; Torensma et al., 2016), is used in practice. The muscle relaxants used in the induction phase of clinical or veterinary anesthesia minimize the voluntary and involuntary excitation and struggling that would otherwise be observed during the excitement phase of anesthesia (Guedel, 1937).

Human beings are conscious and sentient beings and the same is assumed to be true in other advanced vertebrates. Thus, it is often assumed that the clinical anesthetics used to diminish human pain and suffering have similar effects on other vertebrates, mammals in particular, and assumptions about their level of anesthesia are based on their behavioral responses (Guedel, 1937). This is not so easy to verify in invertebrate animals which vary enormously in both physical and behavioral complexity and Varner (2012) proposed that invertebrates, apart from cephalopods, do not feel pain. However, there is substantial evidence that advanced crustaceans may feel pain (Elwood, 2012) based on behavioral and physiological responses to noxious stimuli (McGee and Elwood, 2013) and an electrophysiological investigation of methods of complete anesthesia in lobsters and crayfish was recently carried out (Fregin and Bickmeyer, 2016). It may well be that the precautionary principle should be adopted when evidence for sentience is inconclusive for a particular species (Birch, 2017) and under those circumstances the animal should be considered as sentient until proved otherwise.

The presence of nociceptors does not necessarily mean that animals feel pain because pain is presumed to be an emergent property of the conscious brain (Mather, 2008; Fiorito et al., 2014). However, if advanced invertebrates feel pain this suggests that they must have a degree of consciousness and some appreciation of self. This leads us to the question of what is consciousness? At what level of neural complexity do creatures become conscious of themselves as individuals? We cannot yet answer the first of these questions, but clues are beginning to emerge about consciousness in bilaterians other than in deuterostomes such as advanced chordates, and also in the lophotrochozoan cephalopod molluscs (Godfrey-Smith, 2016; Carls-Diamante, 2017) and in the ecdysozoan decapod crustaceans (McGee and Elwood, 2013). If members of these three disparate animal groups are demonstrably conscious and sentient, but with different neurological structures, we need to determine how they converge to generate self-awareness, but we have not yet reached that position. If we are to do so we will need to determine the common characteristics in the brains of advanced molluscs, arthropods and ourselves. To do this it will be necessary to combine elements of neurophysiology and neuroethology with those from cognitive science and to attempt to understand the emergent properties of neural networks at many “levels above the single neuron” (Bullock, 1958). In common with vertebrates, cephalopod molluscs and arthropods in particular exhibit the following common characteristics:

• condensed central ganglia often organized into a central brain centers due to cephalization during evolution. Thus their nervous systems are hierarchically organized with localization of function (Bullock et al., 1977, chapter 1; Sidorov, 2012), but it should be pointed out that the arms of *Octopus vulgaris* contain about two thirds of the 500 million neurons in the nervous system (Sumbre et al., 2001, 2005, 2006) and are semi-autonomous (Yekutieli et al., 2005a,b; Hochner, 2012)
• complex behaviors, problem solving abilities, play like behavior, learning and memory capabilities, and adult neurogenesis (Wells, 1978; Mather, 1991; Boal, 1996; Kuba et al., 2003; Mather, 2008; Gutnick et al., 2011; Hochner, 2013; Di Cosmo and Polese, 2014; Godfrey-Smith, 2016; Bertapelle et al., 2017)
• possess nociceptors and may have a capacity to feel pain (Crook et al., 2013; Alupay et al., 2014; Burrell, 2017).

The implication is that such animals are all likely to feel pain and recent progress in animal welfare legislation reflects this situation with an increased interest in invertebrate welfare (UK Statutory Instruments, 1993; Sharman, 2004; Moltschaniwskyj et al., 2007; European Parliament and European Union, 2010; Elwood, 2011; De Lisa et al., 2012; Andrews et al., 2013; Horvath et al., 2013; Magee and Elwood, 2013; Fiorito et al., 2014; Polese et al., 2014). Thus, it is now imperative that experimental biologists should pay attention to reducing pain and suffering at least in cephalopod molluscs and decapod crustaceans. For this reason we present this review of the actions of local and general anesthetics, mainly on gastropod molluscs, and also the limited available data on cephalopods, with a view to developing improved anesthetic techniques for cephalopods in the future.

## What is an Anesthetic?

As can be seen from **Table 1**, a wide variety of substances have been used as “anesthetics” on gastropod molluscs in the past, largely prior to the introduction of the more common, non-flammable halogenated ethers, and modern systemic anesthetics and analgesics used clinically and in veterinary care. Thus, many substances have been used as “anesthetics,” but the most appropriate should have relatively few side effects, rapid actions and be rapidly reversible.

**Table 1:** A selection of substances used to relax gastropod species prior to surgery or fixation.

Species used	Type of preparation	Substances used as “anesthetics” or “analgesics”	Investigation	Reference
Muricidae (predatory marine gastropods)	Intact animals	Cocaine	Methods for maximal relaxation before fixation or dissection	Lo Bianco and Hovey, 1899
Amnicolidae, freshwater gastropods	Intact animals	Menthol crystals	Method for full relaxation of animals, followed by morphological studies and histological fixation	Berry, 1943
*Pomatiopsis* spp.	Intact animals	Nembutal for general use but menthol was only partially successful	Relaxing agent for molluscs	Van Der Schalie, 1953
*Physa* spp, *Bulinus* spp.	Intact animals	Menthol and Chlorohydrate (Gray’s mixture)	Relaxation of snails before fixation or dissection	Van Eeden, 1958
*Lymnaea palustris, Physa gyrina, Lymnaea humilus, Pomatiopsis lapidaria, Pomatiopsis cincinnatiensis*	Intact animals	Menthol for small snails; Nembutal for large snails	Relaxation of snails before fixation or dissection	McGraw, 1958
Muricidae	Intact animals	CO2, tetraethyl monothionpyrophosphate, Sevin (1-naphtyl *N*-methylcarbamate)	Method for full relaxation of animals, followed by morphological studies and histological fixation	Carriker and Blake, 1959
*Lymnaea stagnalis*	Intact animal	Nitrogen (to remove oxygen from solution), Nembutal (sodium pentobarbitone), MS222, carbon dioxide	A rapid method for anesthetization and recovery	Lever et al., 1964; updates a paper by Joose and Lever, 1959
Various marine and freshwater gastropods including *Lymnaea stagnalis*	Intact animals	Urethane, ether, MgCl2, propylene phenoxetol (1-phenoxy-2-propanalol), a mixture of Nembutal and MS222 were recommended for internal operations, particularly on *Lymnaea*	Methods for anesthetizing gastropods	Runham et al., 1965
*Agriolimax reticulatus*	Intact animal	Carbon dioxide	Explantation of organs	Bailey, 1969
*Helisoma trivolvis*	Intact animal	Menthol	Preparation for surgical implantation to study functional regeneration of identified neurons	Murphy and Kater, 1980
*Lymnaea stagnalis*	Intact animal and semi-intact preparation	Menthol	Anesthesia of animals prior to dissection. Neurons become quiescent, blockage of chemical synaptic transmission, but not electrical synapses. Probable blockage of Ca^2+^ components of action potential.	Haydon et al., 1982
Snails from the genera: *Biomphalaria*, *Helisoma, Bulinus, Lymnaea, Gyraulis, Anisus*, but not including *Lymnaea stagnalis*	Intact animals	Two variants of the combined use of Nembutal and MS222	Anesthesia prior to injections or transplantation, implantation of various organs	Mutani, 1982
*Helisoma duryi*	Intact animal	Sodium pentobarbital (Nembutal)	Preparation of animal for surgery	Kunigelis and Saleuddin, 1984


## Actions of Clinical Anesthetics on Gastropod Molluscs

Studies on gastropods may well give clues as to the effects of anesthetics on cephalopods, such as *Octopus vulgaris*, given their possible evolution from a common ancestor (Morton, 1958; Moroz, 2009; Shigeno et al., 2010), probably during the Cambrian period (Boyle and Rodhouse, 2005). This being so, findings on the actions of anesthetics on gastropods should give some clues as to their likely effects on cephalopods.

Early studies on sedation of gastropods used a variety of compounds (see examples in **Table 1**), but it is noticeable that menthol (Berry, 1943; McGraw, 1958; Van Eeden, 1958) and Nembutal (sodium pentobarbitone) (Van Der Schalie, 1953; Lever et al., 1964; Runham et al., 1965; Mutani, 1982; Kunigelis and Saleuddin, 1984) have been used alone or in combination with other substances for quite some time. Most of these studies were concerned with ensuring that the animals were sufficiently relaxed for fixation or dissection rather than with animal welfare. In later studies, the actions of anesthetics/analgesics on central nervous function were being considered (e.g., Haydon et al., 1982).

It can prove difficult to specify the differences of response to anesthetics of particular neuron types in vertebrates, given the multiplicity of neurons in the CNS, although modern techniques in electrophysiology, particularly patch clamp, applied to brain slices and isolated neurons have gone some way to alleviating the problem. However, gastropod nervous systems such as those of *Aplysia* (e.g., Chalazonitis and Takeuchi, 1966; Chalazonitis et al., 1966; Ascher et al., 1976; Just and Hoyer, 1977; Marty, 1978; Cote and Wilson, 1980; Arimura and Ikemoto, 1986; Ikemoto, 1986; Ikemoto et al., 1988; Komatsu et al., 1996; Winegar et al., 1996; Winegar and Yost, 1998), *Helix* (e.g., Chalazonitis et al., 1966; Chalazonitis, 1967; Judge and Norman, 1982; Akaike et al., 1982) and *Lymnaea* provide us with excellent models for studies on anesthesia given their large identifiable nerve cells and well-studied behavioral repertoires (e.g., Kandel, 1976; Benjamin, 2012; Winlow and Polese, 2014). Since the middle 1980s a substantial body of work has accrued on the pond-snail *Lymnaea stagnalis* (L.) (e.g., Cruickshank et al., 1985b,c; Franks and Lieb, 1988; Girdlestone et al., 1989a,b; Winlow et al., 1992, 1998; McKenzie et al., 1995; Spencer et al., 1995, 1996; Lopes et al., 1998; Hamakawa et al., 1999; Onizuka et al., 2005b; Browning and Lukowiak, 2008; Onizuka et al., 2008a,b, 2012a,b; Yar and Winlow, 2016; Qazzaz and Winlow, 2017; Armstrong et al., 2018) and on related molluscs (*Euhadra* - Onozuka et al., 1993; *Bulla* – Khalsa et al., 1995; *Achatina fullica* – Lin and Tsai, 2005; Lin et al., 2010; *Tritonia diomedea –*Wyeth et al., 2009; *Elysia viridis* – Cruz et al., 2012). Cephalopod molluscs are of course more complex animals than gastropods and have not easily lent themselves to the sorts of study outlined above. However, a recent major breakthrough has established a new neuronal cell culture protocol for *Octopus vulgaris* (Maselli et al., 2018) and promises to allow more detailed studies at a cellular and subcellular level, including direct studies on the actions of anesthetics on Octopus neurons.

### Gastropods as Model Systems for Anesthetics Research

**FIGURE 1:**
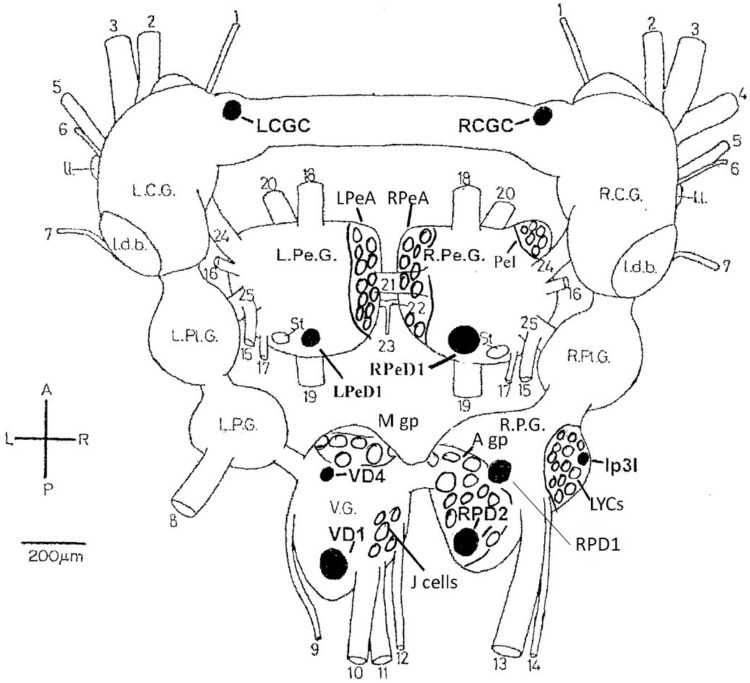
Dorsal view of the central nervous system of *Lymnaea stagnalis* (L.) with the exception of the paired buccal ganglia. The diagram shows locations of individual neurons (black), cell groups and clusters (clear) identified in the text. LCGC and RCGC, left and right cerebral giant cells; RPeD1, right pedal dorsal cell 1; LPeD1, left pedal dorsal cell 1 VD1 and VD4, visceral dorsal cells 1 and 4; RPD1 and RPD2, right parietal; dorsal cells 1 and 2; Ip3I, input 3 interneuron; A gp, right parietal A group; M gp, visceral M group; LPeA and RPeA, left and right pedal A group cells; PeI, pedal I group cells. A, anterior; P, posterior; L, left; R. right. L.C.G. and R.C.G, left and right cerebral ganglia; L.Pe.G. and R.Pe.G., left and right pedal ganglia; L.Pl.G. and R.Pl.G., left and right pleural ganglia; L.P.G. and R.P.G., left and right parietal ganglia: V.G., median visceral ganglion; Idb., lateral dorsal body; II., lateral lobe; St, statocyst. (1) cerebro-buccal connective: (2) superior labial nerve; (3) median labial nerve; (4) penis nerve; (5) tentacle nerve; (6) optic nerve; (7) nuchal nerve; (8) left parietal nerve; (9) cutaneous pallial nerve; (10) intestinal nerve; (11) anal nerve; (12) genital nerve; (13) right internal parietal nerve; (14) right external parietal nerve; (15) inferior cervical nerve; (16) superior cervical nerve; (17) columellar nerve; (18) superior pedal nerve; (19) inferior pedal nerve; (20) medial pedal nerve; (21) dorsal pedal commissure; (22) ventral pedal commissure; (23) medial columellar nerve; (24) cerebro-pedal connective: (25) pedal-pleural connective.

In common with many other gastropods, *Lymnaea stagnalis* (L.), with its large uniquely identifiable nerve cells (Winlow and Benjamin, 1976; Benjamin and Winlow, 1981; Slade et al., 1981) (**Figure 1**), makes studies on the cellular, subcellular, network and behavioral actions of anesthetics more feasible than in either vertebrates or cephalopods. Given that *Lymnaea* has a wide range of neurotransmitters similar to those in cephalopods (Tansey, 1979; Di Cosmo et al., 2004, 2006; D’Aniello et al., 2005; Moccia et al., 2009) this makes it a useful testbed for preliminary studies on more advanced molluscs. What is more, *Lymnaea* has a well-researched behavioral repertoire (Winlow and Polese, 2014) so that both the cellular and behavioral actions of anesthetics can be studied. Central pattern generators (CPGs) for feeding (Benjamin, 2012) respiration (Syed et al., 1990; Syed and Winlow, 1991), locomotion and control of pedal cilia via the Pedal A (PeA) cluster neurons (Syed et al., 1988; Syed and Winlow, 1989) and control of rhythmic shell movements (Haydon and Winlow, 1986) have been reviewed elsewhere (Winlow and Polese, 2014). Application of volatile anesthetics can reveal the properties of neurons within the CPG and can relate those changes to behavioral outputs (McCrohan et al., 1987; Woodall et al., 2001). In *Aplysia californica*, enflurane was found to exert both facilitatory and suppressive control over the rhythmic contractions of the gill and siphon when superfused over the abdominal ganglion and a depolarizing shift in gill withdrawal motor neurons was observed (Komatsu et al., 1993). Similar data has been revealed in vertebrate preparations using isoflurane (Jinks et al., 2005) and opioids (Blivis et al., 2007). Further studies by Komatsu et al. (1996), using a semi-isolated preparation showed that enflurane could have a dual effect, most usually suppressing the gill withdrawal reflex, but in other cases facilitating it, suggesting greater complexity in the underlying neural network than previously reported.

Identifiable, isolated neurons in culture are suitable models for studying the cellular and molecular mechanisms of anesthesia under strictly controlled conditions without the intervention of other neuronal elements (Spencer et al., 1995, 1996; Hamakawa et al., 1999). Cultured *Lymnaea* neurons retain their normal action potential types (Yar and Winlow, 1991; Winlow et al., 1991), transmitter identity (Syed et al., 1990; Spencer et al., 1995; Naruo et al., 2005), responsiveness to applied transmitters (Haydon, 1989; Syed et al., 1990) and responsiveness to applied general anesthetics (Spencer et al., 1995, 1996). Substantial research on the effect of anesthetics on neurotransmission in *Lymnaea* was accrued in Syed’s laboratory in Calgary.

### Pathways to Silence

**FIGURE 2:**
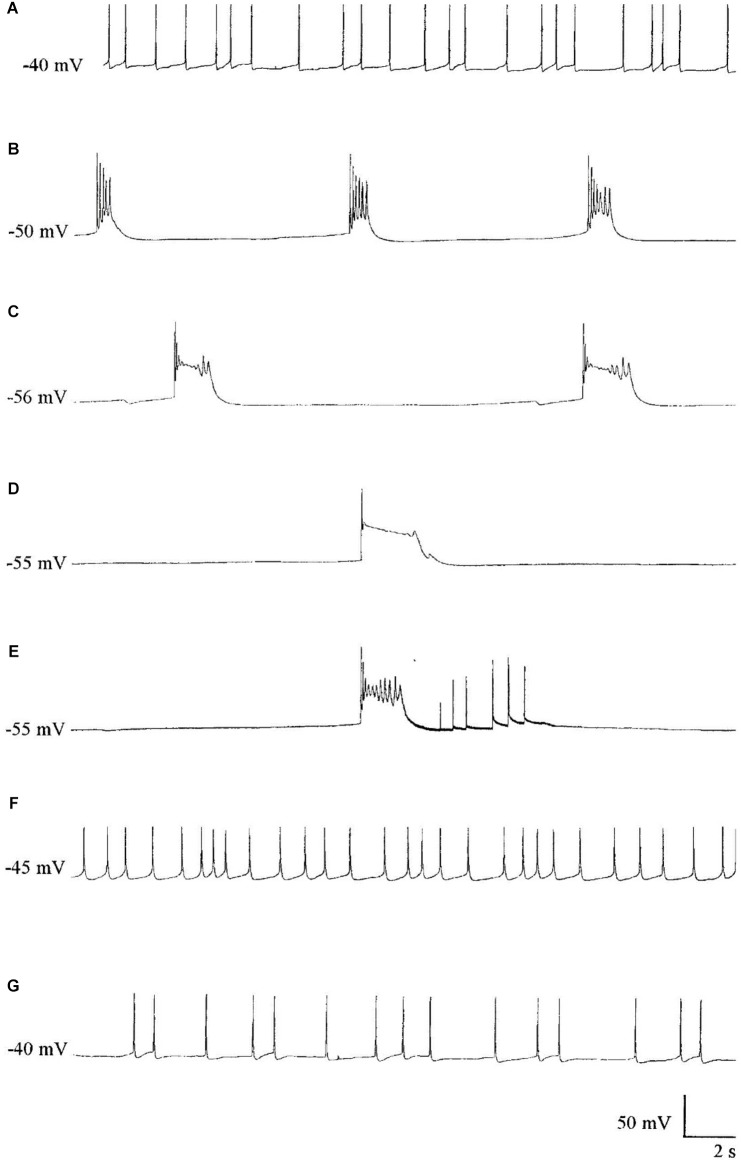
The effect of Pentobarbital on the spontaneous firing pattern and frequency of an A group neuron. **(A)** control; **(B)** 1 mM pentobarbital after 6 min; **(C)** 1 mM pentobarbital after 30 min; **(D)** 2 mM pentobarbital after 6 min; **(E)** 10 min wash out prior to quiescence and **(F)** 30 min wash out and **(G)** 60 min wash out. Membrane potential increased in response to pentobarbital, and decreased as continuous wash out of pentobarbital proceeded. (From Moghadam, 1996 – Reproduced under the Creative Commons License).

Halothane, menthol, ketamine, and also sodium pentobarbitone (Nembutal), which is a sedative often used as a veterinary anesthetic, had differential effects on identifiable neurons in *Lymnaea* (McCrohan et al., 1987; Franks and Lieb, 1988; Winlow et al., 1992). In *Lymnaea*, some neurons gradually became quiescent, whilst others exhibited a series of paroxysmal depolarizing shifts (PDS) (**Figure 2** and **Table 2**) prior to quiescence. Eventually all neurons became quiescent (Winlow et al., 1992) except for the tightly electrically coupled neurons VD1 and RPD2 which become silent in halothane and isoflurane, but exhibit PDS after administration of sodium pentobarbitone. (Qazzaz and Winlow, 2015) (**Table 2**). Evidence now suggests that PDS may be due to suppression of calcium activated potassium currents as a consequence of blockade of voltage gated potassium currents which then unmask persistent sodium currents (Pathak, 2017). Such calcium and potassium currents are known to be affected by general anesthetics in *Lymnaea* (see Systemic General Anesthetics below). Differential effects of Propofol and sevoflurane have recently been demonstrated in the ventrobasal thalamus of mice (Kratzer et al., 2017) suggesting that the loss of consciousness associated with different anesthetics are drug and pathway specific in mammals.

**Table 2:** Differential actions of halothane and sodium pentobarbitone on specific cells and cell groups (see **Figure 1**) in the isolated brain of *Lymnaea.*

	PDS				Quiescence		
Cell type	A gp	J cells	M gp	VD1/ RPD2	RPeD1	RPD1	VD1/RPD2
Halothane (n)	8	2	4		4	2	8
Na Pentobarbitone (n)	10	8	13	10	13	11	


*Data from Girdlestone (1986) and Moghadam (1996). *n*, number of cells studied in each case. With the exception of VD1/RPD2, isoflurane did not cause PDS in the other cell types listed above*.

### Menthol as an Anesthetic

In preliminary behavioral studies on *Lymnaea* a saturated solution of menthol (which is only slightly water soluble) in snail saline (Benjamin and Winlow, 1981) was successfully used as an anesthetic. It had previously been used by Murphy and Kater (1980) to anesthetize the related freshwater snail *Helisoma trivolvis* prior to surgical manipulations, but for experimental work on the central control of behavior it was important to know when the animal had fully recovered from its effects on central identified neurons. This occurred within 40–60 min of washing in normal snail saline (Haydon et al., 1982). In many molluscan neuronal somata inward currents are carried by both sodium and calcium ions (Type 2 action potentials) and menthol suppressed the pseudoplateau during repolarization in these cells. The pseudoplateau is known to be generated by and inward calcium current and appeared to be blocked by menthol. Menthol is now known to have analgesic properties mediated by K-opioid receptors (Galeotti et al., 2002) in mice. It has also been shown to block voltage-dependent sodium channels in rat neurons and human skeletal muscle (Haeseler et al., 2002), where it is said to be as potent as the local anesthetic lidocaine. According to Watt et al. (2008) it shares anesthetic properties with propofol, by its action on GABA_A_ receptors and more recently Lau et al. (2014) have shown it to modulate GABA_A_ – mediated tonic currents and associated inhibitory postsynaptic currents (IPSCs) in rat periaqueductal gray neurons.

### Volatile General Anesthetics in Clinical Use

Over short period from 1956, when halothane was clinically introduced, the non-flammable volatile anesthetics (Terrell, 2008), which are halogenated ethers, gradually replaced flammable volatiles such as trichloroethylene, diethyl ether and cyclopropane previously in clinical use. Volatile anesthetics are toxic, relatively insoluble, with a low therapeutic index and it is essential to do experimental work in the clinically useful range. For example, halothane is much more soluble in blood with a blood/gas partition coefficient of 2.3 compared with isoflurane, 1.4, and so induces anesthesia less quickly, because, in vertebrates, inhalational agents with low solubility in blood diffuse from the alveoli into the circulation more quickly using smaller quantities of the anesthetic (Harvey and Champe, 1992). However, the effects of volatile agents are more easily controllable than systemically applied anesthetics.

Prior to consideration of the actions of volatile anesthetics on isolated brains (Girdlestone et al., 1989a) and cultured identified neurons (Spencer et al., 1995, 1996), it should be noted that volatile anesthetics are usually applied at body temperature in the operating theater. How then does their application at room temperature affect their concentration, since these volatile compounds are more soluble at lower temperatures than at body temperature? Experiments in Winlow’s laboratory were all carried out in the clinical concentration range by careful adjustment of the delivery system combined with measurement of anesthetic concentrations in the experimental dish. A detailed discussion of this issue has been published elsewhere (Qazzaz and Winlow, 2015; Yar and Winlow, 2016), but in short, the work on volatile anesthetics indicates that *Lymnaea* is anesthetized within the clinical range at room temperature.

In order to test whether *Lymnaea* was responsive to modern volatile anesthetics in the clinical range, the whole animal withdrawal response was chosen for study because, as with other animals, protective and escape responses sit at the top of the behavioral hierarchy (Winlow et al., 1992; Winlow and Polese, 2014). Volatile anesthetics are regularly used surgically and their methods of application are well tried and tested. Although in mammals they are delivered via the respiratory system, they are eventually dissolved in body fluids in the lungs and blood stream and therefore the volatile agents were delivered at room temperature direct to the bathing medium of the pond snail. *Lymnaea* respires through a primitive lung via the pneumostome and also across its entire body surface (Mill, 1972) and anesthetics are directly absorbed into the animal from the bathing medium. Experiments with halothane, enflurane (introduced in 1966) and isoflurane (introduced in 1972) showed that the whole-body withdrawal response of *Lymnaea* was dose-dependent within the same clinical range of anesthetic concentrations as man, other mammals, toads and goldfish (Girdlestone, 1986; Girdlestone et al., 1989b). Further experiments on *Lymnaea* have since been carried out successfully with sevoflurane (introduced in 1990) by Morris (1997) and by Syed and his co-workers Thus modern volatile anesthetics are known to be effective in *Lymnaea*, within the clinical range, suggesting that their actions can be generalized to invertebrates.

### Effects of Volatile Anesthetics on Membrane Currents

The general findings on the actions of volatile anesthetics on *Lymnaea* and other gastropods are similar to those in other organisms.

#### Calcium Currents

**FIGURE 3:**
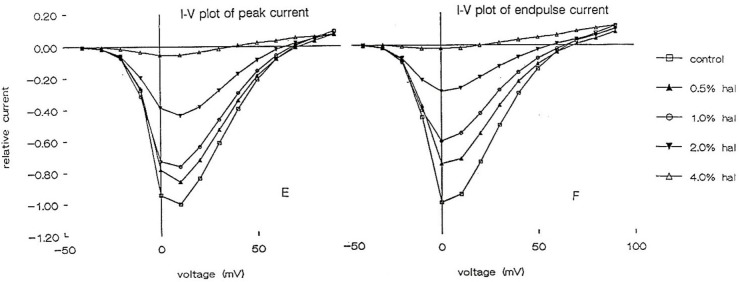
Dose dependent depression of peak current and endpulse current in a PeI cluster cell of *Lymnaea stagnalis.* The Cell was held at a holding potential of –50 mV and 180 ms long depolarizing pulses were applied in 10 mV increments in control (snail saline), 0.5, 1.0, 2.0, and 4.0% halothane. There is a very clear reduction of calcium channel current which is concentration-dependent. The peaks of the I-V curves are not shifted by halothane. (From Yar and Winlow, 2016 – Reproduced under the Creative Commons License).

Using the whole cell clamp technique halothane has been shown to depress high-voltage activated calcium currents and potassium currents in cultured identified *Lymnaea* neurons which are known to retain their normal action potential types (Yar and Winlow, 1991; Winlow et al., 1992). Intracellular recordings from these neurons showed that 1% halothane diminished action potential amplitude, pseudoplateau and after hyperpolarization all of which are calcium dependent phenomena. The effects of four concentrations of halothane were studied on the macroscopic, high-voltage activated calcium channel currents of cultured neurons of the pedal I (PeI) cluster of *Lymnaea stagnalis* (Yar and Winlow, 2016). Following application of increasing concentrations of halothane, in the clinical range, a rapid, reversible and dose-dependent depression of both the peak and end-pulse Ca^2+^ channel currents was observed (**Figure 3**). The rate of inactivation of the calcium current was significantly accelerated by halothane in a dose-dependent manner suggesting that halothane affects the channels both in the open and the closed state. The observations of depression of chemical synaptic transmission, alteration of rate of firing, and alteration in the action potential amplitude, duration and after-hyperpolarization can all be partially explained on the basis of effects of halothane on calcium channels, but volatile anesthetics appear to have multiple targets at the cellular and subcellular levels. Further experiments with isoflurane indicated that low concentrations (0.5%) of isoflurane enhanced the high voltage activated (HVA) current whereas 2% isoflurane significantly decreased it (Moghadam, 1996). In addition, halothane is more potent than isoflurane in decreasing Ca^2+^ currents (Yar and Winlow, 1993). In experiments on the neurosecretory light yellow cells (LYC) (Wendelaar Bonga, 1970; Benjamin and Swindale, 1975) of *Lymnaea*, the effects of sevoflurane were compared with those of halothane and isoflurane on HVA calcium currents (Morris, 1997), revealing that halothane had the most potent effect on peak and end-pulse currents compared with isoflurane and sevoflurane was the least effective.

#### Potassium Currents

**FIGURE 4:**
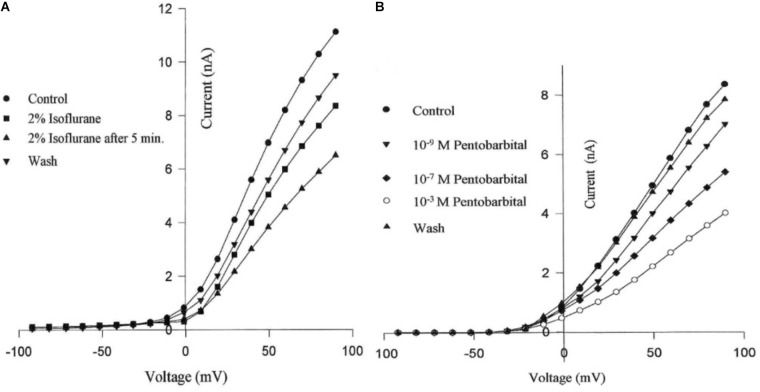
Suppression of potassium currents by both isoflurane **(A)** and sodium pentobarbital **(B)** in cultured pedal I cluster neurons of *Lymnaea*, using the whole cell voltage clamp technique. (From Moghadam, 1996 - Reproduced under the Creative Commons License).

Molluscan neurons have a complicated pattern of outward currents (Reuter and Stevens, 1980) which can be separated either kinetically or pharmacologically (Meech and Standen, 1974; Heyer and Lux, 1976; Thompson, 1982). K^+^ channels are divided into two categories; those activated by the membrane potential and those activated by various modulators (Schwarz and Passow, 1983). Pharmacological separation of K^+^ currents on isolated identified PeI cluster cells of *Lymnaea* in culture using the whole cell clamp technique demonstrated that several potassium currents could be identified in PeI cluster neurons: transient current (A current), K_ATP_, Ca^2+^ dependent K^+^ current (I_KCa_) and the delayed rectifier current (I_k_). The ATP dependent current, K_ATP_, has a low open state probability and is regulated by intracellular ATP and other metabolites (Nelson et al., 1990; Larach and Schuler, 1993). Pentobarbital significantly decreased K_ATP_, I_KCa_ and I_k_ in a dose dependent manner (**Figure 4B**) (Moghadam and Winlow, 1995b; Winlow et al., 1995; Moghadam, 1996). Both halothane and isoflurane (**Figure 4A**) depressed gross K^+^ currents (Moghadam and Winlow, 1995a), but halothane is more effective than isoflurane in depressing the gross K^+^ current of PeI cluster neurons. Furthermore, halothane partially depressed I_KCa_ as do other anesthetics (Moghadam, 1996). However, many different cell types exist within the nervous system of *Lymnaea* and Lopes et al. (1998) demonstrated the unexpected, but significant finding, that volatile anesthetics activated the novel potassium current I_K(AN)_ first described by Franks and Lieb (1988). This current was not demonstrable in the surrounding Light Yellow Cells (LYCs) (Wendelaar Bonga, 1970; van Swigchem, 1979) in the right parietal ganglion. Similar K^+^channels - the serotonin activated S channels - have been observed in isolated, cultured neurons from *Aplysia californica* using patch clamp techniques (Winegar et al., 1996) when treated with halothane or isoflurane. In addition I_K(AN)_ is thought to be activated by certain cytrochrome P450 isoforms (Lopes et al., 1998).

#### Sodium and Chloride Currents

Many gastropod action potentials have both sodium and calcium components (Type 2 action potentials) as in the giant *Aplysia* neuron R2 from the abdominal ganglion (Geduldig and Junge, 1968) and various neurons from *Helix* (Gerasimov et al., 1964, 1965) and *Lymnaea* (Haydon et al., 1982; Winlow et al., 1982). Chalazonitis (1967) considered the depressive and selective effects of halothane, ether and chloroform on identifiable neurons of *Helix* and *Aplysia* and showed that their somatic action potentials were much more sensitive to volatile anesthetics than were axonal action potentials. Later, Geduldig and Junge clearly demonstrated that both sodium and calcium currents contributed to somatic action potentials of the Aplysia R2 but that the axon spike was only sodium dependent (Junge and Miller, 1974).

### Systemic General Anesthetics

Although volatile anesthetics are easier to control than systemic anesthetics, systemic anesthetics may, under some circumstances, be easier to administer. Some of the intravenous anesthetics cause irritation and pain on injection or hangover after recovery or have the potential to produce allergic reactions and histamine release or several effects on the cardiopulmonary system (Reilly, 1994). The ideal intravenous anesthetic would of course induce hypnosis, amnesia and analgesia (Reves and Glass, 1990).

Barbiturates are not stable in solution for more than 24 h (Reilly, 1994) and have relatively short actions (ultrashort acting barbiturates) (Goth, 1976). In addition to anesthesia some of the barbiturates also induce convulsions (Daniell, 1994). Pentobarbital was used for many years as a soporific and anesthetic agent (O’Beirne et al., 1987) and has been suggested to be suitable for anesthesia of *Biomphalaria tenagophila* and *B. glabrata* (Martins-Sousa et al., 2001). Thiopentone is a potent anesthetic which induces anesthesia in seconds, but postanesthetic care is required. Large doses of thiopentone given clinically induce circulatory depression and even small doses cause central respiratory depression (Goth, 1976). Phenobarbitone and barbitone are long-acting barbiturates and are less lipophilic than ultra-short and short-acting barbiturates such as thiopentone and pentobarbital (Bush, 1963). Some of the effects of anesthetics can be accounted for by their solubility in the membrane matrix which causes conformational changes to ionic channels, in accord with the observations of Judge et al. (1979) on identified neurons in the brain of *Helix aspersa*, that thiopentone may have a non-selective action on receptor-coupled ionophores rather than on specific receptors for acetylcholine, glutamate and dopamine and Somei (1981).

Frequency-dependent spike broadening is a characteristic feature of molluscan neurons with mixed sodium/calcium dependent somatic action potentials and pentobarbital and phenobarbitone reduce action potential duration with sedative and anesthetic doses in large multipolar spinal cord neurons (Heyer and Macdonald, 1982). Pentobarbital can reduce L and N type Ca^2+^ currents in a dose dependent manner in cultured mouse neurons (Gross and Macdonald, 1988), and thus might reduce frequency, width and AHP of the action potential. Furthermore, the anticonvulsant/hypnotic agent, phenobarbital, appears to act through depression of frequency-dependent spike broadening on neurons of *Helix aspersa* (Eagan et al., 1987) which could in turn depress excitatory transmission at nerve terminals, possibly a general mechanism for barbiturate actions, and supported by observations of the effects of pentobarbital on *Aplysia* neurons (Ikemoto et al., 1986). It has been suggested that the sodium and calcium currents underlying the spikes are equally sensitive to pentobarbital (Goldring and Blaustein, 1982) in the giant R2 neuron of *Aplysia.* Thiopentone, pentobarbitone, phenobarbitone and barbitone, all accelerated the decay phase of the I_Ca_ in *Helix aspersa* neurons (Nishi and Oyama, 1983a), and pentobarbitone also inhibited its maximum peak amplitude (Nishi and Oyama, 1983b). In *Aplysia* neurons in excised ganglia, both pentobarbital and phenobarbital enhanced spike frequency adaptation via a slowly developing outward current unique to neurons of this type (Cote et al., 1978; Zbicz and Wilson, 1981) and in other neurons they depressed chloride-dependent inhibitory responses to either iontophoretically applied acetylcholine or GABA (Cote and Wilson, 1980) and attenuated excitatory responses while potassium-dependent inhibitory responses were minimally affected. However, patch clamp recordings from the circadian pacemaker cells of the *Bulla* eye showed that pentobarbital reduces the calcium dependent potassium current, probably by reducing an inward calcium current (Khalsa et al., 1995).

The after hyperpolarization (AHP – also termed: SK channels, slow AHP, K_Ca_, I_AHP_ and the apamin sensitive calcium-activated potassium channel after the bee venom neurotoxin (Moczydlowski et al., 1988; Strong, 1990a,b), which is a Ca^2+^ dependent phenomenon, was reversibly decreased or abolished with pentobarbital (Moghadam, 1996). Furthermore, different AHPs exist in different cell types in *Lymnaea* with respect to their sensitivity to pentobarbital and time dependency (onset of action). The AHP, which occurs after an action potential and is due to a Ca^2+^ activated K^+^ conductance (Hoston and Prince, 1980; Schwartzkroin and Stafstrom, 1980), decreased significantly in those *Lymnaea* neurons exhibiting PDS in response to pentobarbital applications (Moghadam, 1996) (**Table 2**). For example, the AHP in M group neurons disappeared after 24 min, and then PDSs appeared during continuous pentobarbital application. This phenomenon suggests that the response of components of the AHP in these neurons is time dependent with respect to pentobarbital application.

1-phenoxy-2-propanol (PP) (aka: propylene phenoxitol) is a glycol ester that has been shown to have fully reversible anesthetic properties on gastropods including *Hermissenda crassicornis, Tritonia diomedea* and *Lymnaea stagnalis* (Wyeth et al., 2009). PP can be bath applied and is biodegradable. It has been used relax or anesthetize molluscs and other groups since 1955 (Owen, 1955; Runham et al., 1965 - see **Table 1**), but its anesthetic actions have only recently been investigated and Wyeth et al. (2009) have demonstrated that it reversibly eliminates neural activity, acts as a muscle relaxant and eliminates behavior. They suggest that it “is a useful candidate for gastropod anesthesia.”

### General Anesthetics Raise Intracellular Calcium Concentration

**FIGURE 5:**
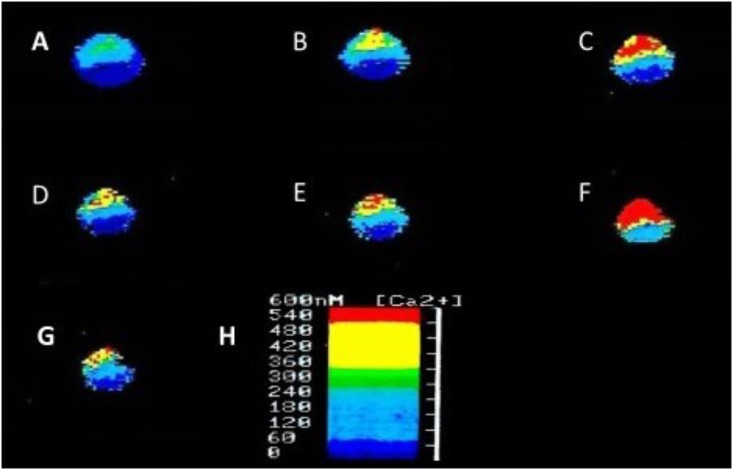
Ratiofluorimetric images showing that Halothane raises intracellular calcium concentration in the cultured *Lymnaea* neurone RPD2 loaded with the fluorescent Ca^2+^ indicator Fura 2. **(A)** Control in normal saline (101.18 nM [Ca^2+^]_I_); **(B)** 1 min after addition of 2% halothane (174.12 nM [Ca^2+^]_I_); **(C)** 5 min after 2% halothane (287.06 nM [Ca^2+^]_I_); **(D)** 10 min after washout of 2% halothane with normal saline (183.53 nM [Ca^2+^]_I_); **(E)** 1 min after 4% halothane (232.94 nM [Ca^2+^]_I_); **(F)** 5 min after 4% halothane (414.12 nM [Ca^2+^]_I_); **(G)** 15 min after washout of 4% halothane with normal saline (211.76 nM [Ca^2+^]_I_); **(H)** Fluorescence scale bar (From Ahmed, 1995 – Reproduced under the Creative Commons License).

**FIGURE 6:**
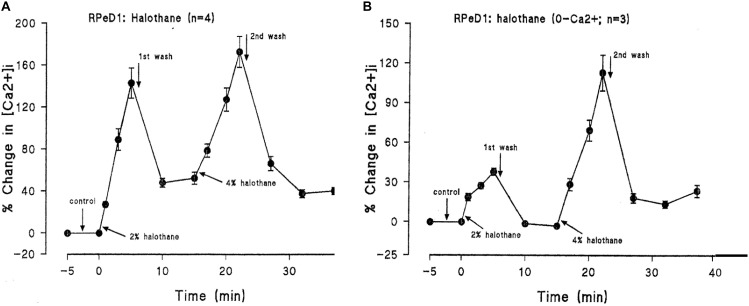
Halothane modifies intracellular calcium concentration in RPeD1, **(A)** when vaporized into normal saline, **(B)** when vaporized into zero calcium saline (From Ahmed, 1995 – Reproduced under the Creative Commons License).

In addition to suppressing both calcium and potassium currents in a dose dependent manner, both halothane and pentobarbitone raise intracellular calcium concentration, [Ca^2+^]_I_ (**Figure 5**), from intracellular sources (Ahmed, 1995; Winlow et al., 1995; Moghadam and Winlow, 2000) also in a dose dependent manner, even in the presence of zero external calcium (**Figure 6**). The increase in [Ca^2+^]_I_ is unlikely to be due to any sodium-calcium exchange effect as it occurs in the absence of external calcium. Because of their lipid solubility anesthetics must have numerous effects both on the plasma membrane and on intracellular sites. Since calcium stores are likely to vary in extent from one neuron type to the next the resulting levels of free [Ca^2+^]_I_ will most likely have effects on membrane permeability as well on other metabolic processes. Similar effects have also been demonstrated in squid axons (Vassort et al., 1986), hippocampal cells (Mody et al., 1991), and mouse whole brain synaptosomes (Daniell and Harris, 1988).

### Actions of General Anesthetics on Synaptic Transmission

#### Chemical Transmission

At chemical synapses neurotransmitter substances are released from nerve cells when a nerve impulse arrives to trigger calcium entry into the synapse, through calcium channels. Experiments on identified cells in the abdominal ganglion of *Aplysia oculifera* suggest that enflurane depresses excitatory and inhibitory cholinergic transmission by reducing postsynaptic currents (Arimura and Ikemoto, 1986), but the postsynaptic responses to ACh were complex: it evoked three non-competitive postsynaptic responses under voltage clamp, a depolarizing response due to increased Na^+^ and K^+^ conductances, a delayed Cl^-^ conductance increase and a slow increase K^+^ conductance, both causing hyperpolarization. Further research showed that halothane had differential actions on glutamate and acetylcholine induced chloride currents in single mechanically isolated, but unidentified, neurons of *Aplysia kurodai*, suggesting that they had different specific binding sites in the receptor-channel complexes (Ikemoto et al., 1988). The anesthetic also resulted in the decay of epsps and ipsps evoked by stimulation of the pleuro-abdominal connective.

Such studies as those set out above have been invaluable, but it can be very difficult to have unambiguous proof that the experimenter is working with monosynaptically connected neurons within the nervous system. Synaptically connected pairs of neurons in culture resolve this problem and are unequivocally known to be coupled together. The original work in this area was carried out in Puschino (Russia) by Kostenko (1972) and Kostenko et al. (1974) and continued in Kater’s laboratory at the State University of Iowa (e.g. Haydon and Kater, 1987) using the pond snail *Helisoma trivolvis.* The neuronal strategies for the formation of chemical synapses were clearly set out by Haydon (1989) where pairs of spherical cell bodies effectively give access to the synaptic terminal. This approach has subsequently been further developed in Syed’s laboratory at the University of Calgary using identified, cultured neurons from *Lymnaea stagnalis*. Using these techniques, it has been shown that VD4 can form peptidergic monosynaptic connections cells of the PeA clusters, first demonstrated in the intact brain and then in culture. In both cases halothane induced synaptic depression of the IPSP between the monosynaptically connected cells and enhanced the postsynaptic inhibitory response to exogenously applied FMRFamide in cell culture (Spencer et al., 1995, 1996). This latter finding indicates that depression of transmission probably occurred presynaptically. PeA cells may receive either inhibitory or excitatory connections from VD4 and excitatory peptidergic transmission between cultured *Lymnaea* neurons is more sensitive to depression by halothane than is inhibitory transmission (Spencer et al., 1996). Halothane induces a novel dose-dependent depolarizing response to met-enkephalin on isolated PeA neurons in place of the hyperpolarization, but after washout there was no further response to applied met-enkephalin (Spencer et al., 1998; Winlow et al., 1998, 2000). The mechanisms underlying these modifications are as yet unknown, but peptidergic transmission is clearly susceptible to the effects of volatile anesthetics.

**FIGURE 7:**
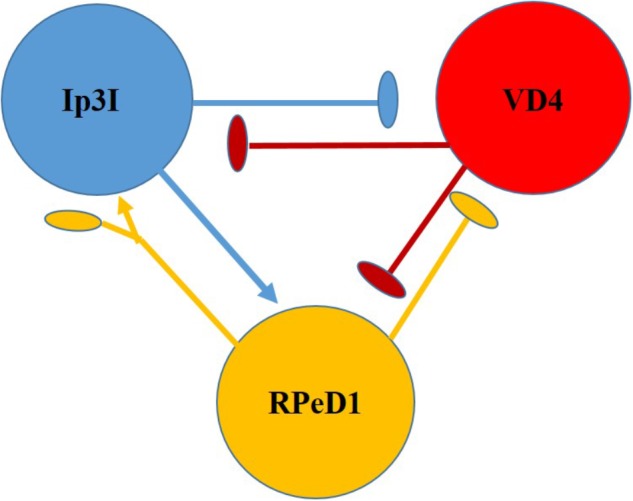
Diagram of the neurons making up the respiratory CPG of *Lymnaea stagnalis.* RPeD1 initiates the RCPG rhythm, VD4 drives inspiration and pneumostome closure, while the input 3 interneuron (Ip3I) drives expiration and pneumostome opening. Ovals are inhibitory synapses and arrows are excitatory synapses. RPeD1 has a biphasic synaptic connection to Ip3I (For further detail, see Syed et al., 1990).

Clinically relevant concentrations of sevoflurane have (1–4%) also been tested on dopaminergic inhibitory synapses between the soma-soma paired *Lymnaea* neurons RPeD1 and VD4 (**Figure 7**) (Hamakawa et al., 1999). RPeD1 ipsps on VD4 were found to be reversibly blocked by 4% sevoflurane and action potentials in both cells were suppressed. Dopamine activated a voltage-insensitive K^+^ current in VD4 which was also induced by sevoflurane and was probably analogous to IK(An) (Franks and Lieb, 1991). Since HVA calcium currents were not significantly depressed in RPeD1, it is probable that the K^+^ induced presynaptic hyperpolarization reduced the presynaptic transmitter release. Sevoflurane also blocks cholinergic epsps between cultured VD4 and LPeD1 reversibly. In this case presynaptic transmitter release was unaffected, but postsynaptic nicotinic receptors were blocked in a dose dependent manner. However post-tetanic potentiation (PTP), a form of working memory, established in the absence of the anesthetic was not eliminated by it (Naruo et al., 2005).

A series of systemic general anesthetics has been applied to identified neurons of *Helix aspersa* to test their action on ACh-induced inhibition using the voltage clamp technique. Thiopentone, etomidate, minaxolone and ketamine all reversibly depressed a chloride-dependent inhibitory response to ACh in a dose-dependent manner, supporting the view that these anesthetics act postsynaptically on receptor coupled ionophores (Judge et al., 1979; Judge, 1980; Judge and Norman, 1980). A group of three or four neurons isolated from the right parietal ganglion of *Lymnaea*, lateral to the light-yellow cells have nicotinic acetylcholine receptors against which McKenzie et al. (1995) tested 31 general anesthetics, observing their half maximum inhibitory concentrations (IC_50_). The inhalational anesthetics were the most potent inhibitors of the ACh receptor and not dissimilar to their anesthetic potencies on tadpoles.

Behavioral studies following injection of propofol and ketamine caused excitation of intact *Lymnaea stagnalis* (Woodall and McCrohan, 2000). In *Aplysia* neurons it has been suggested that the dissociative anesthetic, ketamine, depresses cholinergic responses by affecting gating at postsynaptic membranes (Ikemoto, 1986). Ketamine started to be developed in the 1950s and proved potent in analgesia (Mion, 2017), but prolonged exposure may induce neurotoxicity and neuroapoptosis via the PKC/ERK pathway in cultured hippocampal neurons, while activation of excitatory NMDA receptors reverses these effects (Jiang et al., 2018). In some neural stem cell derived neurons, ketamine neurotoxicity appears to be due to NMDA-receptor mediated calcium influx (Wang et al., 2017). Alcohol is also known to amplify ketamine induced apoptosis in cultured cortical neurons and PC 12 cells by down regulation of CREB-related signaling pathways (Zuo et al., 2017). Given the drawbacks of ketamine as a rapid and sustained anti-depressant, its metabolites and other modulatory agents of NMDA receptors are now being studied with a view to the development of less psychoactive substances (Chaki, 2017).

Ketamine has been shown to inhibit long-term memory formation in *Lymnaea*, probably due to its effects on underlying molecular processes, while leaving intermediate-term memory intact (Browning and Lukowiak, 2008). More recently acutely applied ketamine has been shown to significantly decrease synaptic transmission between paired VD4 and LPeD1as well as electrical transmission between PeA neurons *in vitro*, although not in a concentration dependent manner, but it did not significantly affect short-term synaptic plasticity which is thought to underlying short-term, working memory (Woodruff et al., 2015). Earlier experiments on paired VD4 and LPeD1 *in vivo* with propofol showed that the anesthetic blocked synaptogenesis, but not neuronal regeneration. However, synaptogenesis developed several hours after washout of propofol (Woodall et al., 2003).

#### Electrical Transmission

Electrical synapses are characterized as gap junctions which allow current to flow between cells, but whose conductances may be modulated by chemical synapses or by anesthetics, resulting in modulation of coupling between cells (Winlow et al., 2017). Comparative studies with halothane, isoflurane and pentobarbitone on the isolated brain of *Lymnaea* show that they have very different effects from one another (Winlow et al., 1998; Qazzaz and Winlow, 1999, 2015, 2017). Volatile anesthetics decreased coupling between cells in a dose dependent manner with one notable exception, which requires further research: the very strong electrical connection between the left and right cerebral giant cells whose connection is maintained even in 8% halothane (Girdlestone et al., 1989a). Sodium-pentobarbitone had markedly different effects from the volatile anesthetics. Clinical concentrations of pentobarbitone (100 μM) caused VD1 and RPD2 to become quiescent with no significant change in either their resting membrane potential or the coupling coefficient between them, but at higher concentrations of pentobarbitone both cells exhibited PDS during which the coupling coefficient increased significantly, before quiescence during which the coupling coefficient fell to control values (Qazzaz and Winlow, 2015). A further study on the passive properties of VD1 and RPD2 (Qazzaz and Winlow, 2017) revealed that the differential changes in coupling coefficient were underlain by modifications to input resistance [*R_m_*], membrane time constant [*τ_m_*] and capacitance [*C_m_*] (Qazzaz and Winlow, 2017), presumably due to membrane conductance changes of the type set out in Sections “Systemic General Anesthetics” and “General Anesthetics Raise Intracellular Calcium Concentration” above.

### Local Anesthetics

Research into the actions of local and volatile anesthetics on identified, cultured *Lymnaea* neurons demonstrated that clinical concentrations of lidocaine and bupivacaine suppressed neurite outgrowth and drastically reduced synapse formation between soma-soma paired neurons (VD4 and LPeD1) whereas the inhalation anesthetics sevoflurane and isoflurane allowed both neurite regeneration and synapse formation between these cells (Onizuka et al., 2005a).

#### Cocaine and Its Structural Relations

Local anesthetics are structurally related to cocaine and block voltage-sensitive sodium channels. Most molluscan studies on local anesthetics have been carried out using cocaine itself and procaine, norcocaine, lidocaine, bupivacaine and dubicaine. Norcocaine is a minor metabolite of cocaine and its only confirmed pharmacologically active metabolite. It has been found to have greater potency than cocaine itself when tested against neurons of the visceral ganglion of *Aplysia californica* (Just and Hoyer, 1977) using conventional microelectrode recordings and the phase-plane technique (Winlow et al., 1982; Holden and Winlow, 1984). However, cocaine is still more commonly used. Furthermore, cocaine has been shown to activate an inward calcium current at low concentrations and to inhibit it at higher concentrations in internally perfused neurons of *Lymnaea stagnalis* (Vislobokov et al., 1993). In *Achatina fulica* (Ferussac) cocaine was found to elicit action potential bursts in the identifiable neuron RP4 probably due to its inhibitory effects on the delayed rectifying K^+^ current, in addition to which it decreased I_Ca_, the fast inactivating K^+^ current and I_KCa_ (Chen et al., 2006). In intact *Lymnaea*, cocaine was found to inhibit dopamine uptake in isolated snail brains and to impair the extinction of memory, an active learning process, during reinstatement of operant conditioning of the intact animal (Carter et al., 2006).

##### Lidocaine

Lidocaine (also known as xylocaine and lignocaine) is widely used to block sodium channels to impart pain relief in regional anesthesia and postoperatively, but there are many reports of neurotoxicity following its use in mammals and molluscs, where it can cause an extreme increase in intracellular calcium concentration (Gold et al., 1998; Johnson et al., 2002; Armstrong et al., 2018) and/or damage to plasma membranes (Kanai et al., 2000). Dibucaine and bupivacaine have damaging effects on growth cones (Kasaba et al., 2001). Of seven local anesthetics tested on the morphology of growth cones and neurites of developing, cultured *Lymnaea* neurons, lidocaine was found to be the most toxic (Kasaba, 2007; Kasaba et al., 2003, 2007). Lidocaine has also been found to increase sodium concentration and to promote depolarization and excitation through voltage–dependent sodium channels (Onizuka et al., 2004) of the giant dopamine-containing neuron RPeD1 (Winlow and Benjamin, 1977; Cottrell et al., 1979; Haydon and Winlow, 1981; Winlow et al., 1981), which is part of the respiratory central pattern generator (rCPG) in *Lymnaea* (Syed et al., 1990) (**Figure 7**). Soma –soma pairing of two neurons from the rCPG, RPeD1 and VD4 allowed Onizuka et al. (2008b) to consider the effects of lidocaine on synaptic transmission between mutually inhibitory neurons, both of which were depolarized in a dose dependent manner, resulting in increased spike frequency and action potential broadening as well as dose-dependent decreases in outward potassium currents and inward calcium currents. IPSPs between the two neurons were suppressed at high lidocaine concentrations, promoting excitation of the two neurons. However, lidocaine has also been shown to raise intracellular calcium concentration and to cause morphological damage and shrinkage to cells even in the presence of a calcium chelating agent to prevent the rise in intracellular calcium concentration (Kasaba et al., 2006). This suggests that lidocaine may have a direct effect on cell morphology and shrinkage. Although lidocaine blocks voltage-dependent sodium channels, in common with other local anesthetics such as mepivacaine and prilocaine, it also increases intracellular sodium concentration, [Na^+^]in, via a Na^+^- H^+^ exchanger activated by intracellular acidification (Onizuka et al., 2008a). It is suggested that entry of the base form of lidocaine enters the cell thus causing acidification by trapping protons and causing cellular toxicity. Furthermore, Onozuka et al. (1993) have demonstrated that lidocaine suppression of Na^+^ current in *Euhadra* neurons is mediated by cyclic AMP dependent protein phosphorylation. Clinical doses of lidocaine have been found to cause necrosis and apoptosis in the cultured *Lymnaea* LPeD1 neuron with a dose-dependent decrease in membrane resistance and an increase in membrane capacitance (Onizuka et al., 2012b).

Using reconstructed *Lymnaea* cholinergic synapses from VD4 to LPeD1 it has been possible to demonstrate that lidocaine suppresses excitatory transmission in a dose dependent manner: presynaptically by voltage-dependent inactivation of the presynaptic membrane due to depolarization and postsynaptically by reducing the response to iontophoretically applied acetylcholine (Onizuka et al., 2008b). By pairing VD4 with neurons from the left pedal E (LPeE) cluster it has also been possible to show that lidocaine treatment during synapse formation permanently suppresses nerve growth factor (NGF) induced excitation by suppressing both axonal growth and neurotransmitter release from presynaptic neurons (Onizuka et al., 2012a) probably by suppressing voltage-dependent calcium currents (Kasaba et al., 2006).

##### Procaine

Experiments on small neurons of the pleural ganglia of *Aplysia californica*, have shown that procaine blockade of acetylcholine induced depolarizations, similar to those at the frog neuromuscular junction (Katz and Miledi, 1975), and resembles the effects of curare and hexamethonium. Its actions can be accounted for by assuming that procaine binds preferentially to the activated receptor-channel complex and converts it into a non-conducting state (Ascher et al., 1976; Marty, 1978). In *Achatina fulica* (Ferussac), procaine initiated paroxysmal depolarizing shifts (PDSs) in the RP1 neuron, which were decreased if lithium replaced Na^+^ in the bathing medium or in a high magnesium solution (Lin and Tsai, 2005), suggesting similar effects to those observed with lidocaine by Onizuka et al. (2012a) (see above). In addition, PDS was associated with phospholipase activity and calcium mobilization in the neuron (Lin et al., 2010).

#### Opioids and Opiates

In gastropod molluscs endogenous opioid systems are involved in modulation of nociception and analgesic responses (Kavaliers, 1991; Liu, 2008; Miller-Pérez et al., 2008). At a behavioral level the terrestrial pulmonate *Cepaea nemoralis* has been shown to develop tolerance to morphine induced analgesia (Kavaliers and Hirst, 1986), but required specific environmental cues for the tolerance to be expressed, consistent with a classical conditioning of habituation model. The activity of the serotonergic ciliomotor neurons (PeA clusters) of *Lymnaea stagnalis* (Syed and Winlow, 1989; McKenzie et al., 1998) is suppressed in the presence of the opiate antagonist naloxone resulting in a decrease in the rate of ciliary locomotion, whereas opiate agonists such as morphine accelerated the synaptically driven firing of the PeA neurons *in vivo* (D’iakonova, 1998). Voltage clamp studies by Vislobokov and Savos’kin (2000) on the opioid analgesics morphine, promedol, tramadol, and butorphanol indicate that sodium and potassium currents in *Lymnaea* neurons *in vivo* are reversibly inhibited and non-specific leak currents are reduced, thus stabilizing the cell membrane. It has been suggested that morphine and related compounds decrease the efficacy of a presumed cholinergic epsp on cell R15 in the intact abdominal ganglion of *Aplysia californica* by reduction of transmitter release (Tremblay et al., 1974). However, the vertebrate morphine antagonist naloxone did not inhibit the actions of morphine but decreased the epsp amplitude suggesting that the opiate receptor was not stereospecific. More specific experiments by Waziri (1976) on the identified multiaction neuron L10 and its identified follower cells, some of which it excites and some of which it inhibits, yielded rather different results. The effects of morphine were demonstrated to be postsynaptic by receptor blockade, rather than presynaptic. In *Lymnaea* there is substantial evidence for an enkephalinergic system (Leung et al., 1990), for example the opioid peptide met-enkephalin can modulate membrane potentials and rhythmic activity in its central neurons (Moroz and Winlow, 1993). Met-enkephalin can also enhance electrical coupling between identified neurons in both *Helix pomatia* and *Lymnaea* (Dyakonova et al., 1993) by increasing their input resistance, but these effects of met-enkephalin on the weakly electrically PeA cluster neurons are reversibly abolished by halothane or isoflurane (Qazzaz and Winlow, 1999). Furthermore, the presence of opioid receptors has been proposed in cephalopods by Stefano et al. (1981), and they are believed to play a prominent role in regulation of transmitter release in most invertebrates (Sha et al., 2013).

## Actions of Anesthetics on Cephalopod Molluscs

Given that so much of the early work on the understanding the mechanism of the action potential was carried on the giant axons of the squid *Loligo forbesi* (see review by Hille, 1992, chapter 2) and several closely related species and on synaptic transmission at the squid giant synapse (reviewed by Llinás, 1999), it is most likely that effects of clinical general and local anesthetics on cephalopods are similar to those found in vertebrate and gastropod preparations. The actions of general anesthetics on *Loligo forbesi* have recently been reviewed by Armstrong et al. (2018), but briefly all anesthetics were found to diminish axonal potassium and sodium currents (Shrivastav et al., 1976; Haydon and Urban, 1983, 1986), with a resultant slight depolarization of the membrane (Haydon and Simon, 1988) and recognition of a highly anesthetic sensitive, voltage independent K^+^ conductance in the resting squid axon (Haydon et al., 1988; Elliott et al., 1989; Hendry and Haydon, 1991). The actions of clinical concentrations of different anesthetics had variable effects on action potential threshold (Haydon and Simon, 1988). Experiments with barbiturate anesthetics revealed that both thiopentone and pentobarbital also suppress Na^+^ and K^+^ conductances (Sevcik, 1980) and ketamine appears to suppress both peak transient and steady state Na^+^ conductances and is suggested to induce Na^+^ accumulation inside the cell which might account for reduction of peak transient current (Shrivastav, 1977). With regard to local anesthetic actions on the squid giant axon, lidocaine and its derivatives appear to reduce the Na^+^ current by inactivating a receptor within the Na^+^ channel (Narahashi and Frazier, 1975; Cahalan and Almers, 1979a,b; Bekkers et al., 1984) and the drug molecule is trapped by the activation gate of the channel (Yeh and Tanguy, 1985), in accord with Judge et al. (1979), Judge and Norman, 1980 on gastropods.

The giant fiber system of *Loligo* was first described by Young (1939). Although the system is not found in Octopods, the giant fiber system has proved to be such a good model for the basic working of neural signaling and synaptic communication, there is little doubt that it is also a good basic model for the functioning of the Octopus nervous system, particularly as cephalopods in general provide good models for understanding the neural substrates underlying behavior (Young, 1964; Williamson and Chrachi, 2004).

### Anesthetizing Cephalopods

Ever since 2013 700 species of cephalopods have had the same legal protections as vertebrates under the European Union Directive 2010/63/EU which means, among other things, that “*objective criteria need to be developed to identify signs of suffering, distress and lasting harm particularly in the context of their induction by an experimental procedure*” (Fiorito et al., 2014). Previous attempts to anesthetize cephalopods have used a variety of substances (see Gleadall, 2013) including muscle relaxants such as magnesium chloride (Messenger et al., 1985; Mooney et al., 2010; Gonçalves et al., 2012; Gleadall, 2013; Butler-Struben et al., 2018) and low temperatures (Andrews and Tansey, 1981), resulting in paralysis of the animals rather than anesthesia. Even ethanol has been used for short duration tagging of the oval squid (Ikeda et al., 2009). A recent study by Gonçalves et al. (2012) compares several different agents commonly used to immobilize *Sepia officinalis* and indicates that hypothermia causes severe stress reactions during the recovery phase while recommending that MgCl_2_ is an appropriate “anesthetic” agent causing little stress to the animal under these conditions. However, MgCl_2_ is not an anesthetic agent, and neither of these treatments is acceptable as a form of anesthesia because a loss of consciousness cannot be assumed in the absence movement without detailed analysis of behavioral signs of anesthesia. Additionally, cephalopods are relatively large animals and it is unlikely that immersion in magnesium chloride would have rapid central effects as previously asserted by Messenger et al. (1985). Other muscle relaxants used in cephalopods include gallamine (Flaxedil), and MS-222 (tricaine methanesulfonate or mesylate) (Frazier and Narahashi, 1975; Mooney et al., 2010). MS-222 is used as a muscle relaxant in cephalopods and as an anesthetic and sedative in fish (Palmer and Mensinger, 2004), but has only been shown to act in a similar way to local anesthetics (Frazier and Narahashi, 1975), depressing peak sodium current and steady state potassium currents, but tending to depolarize the axon. While muscle relaxants may have a role in anesthetic induction procedures, none of these substances have anesthetic actions in themselves. Gallamine application to the squid *Doryteuthis peleii* resulted in death of the animals as did the local anesthetic, benzocaine, and the analgesic, clove oil (Mooney et al., 2010). The intravenous anesthetic urethane (ethyl carbamate) was used to anesthetize cephalopods in the past (Messenger, 1968; Young, 1971), but fell into disfavor when found to be carcinogenic and endangered laboratory personnel (Field and Lang, 1988). Ethanol is also unsuitable as it tends to cause the escape behaviors of jetting and inking on initial immersion of the animal (Andrews and Tansey, 1981; Lewbart and Mosley, 2012) although this phenomenon is not always reported (Froesch and Marthy, 1975; Harms et al., 2006). Mooney et al. (2010) described similar phenomena when *D. peleii* were placed in ethanol as well as dramatic color changes not normally seen in the animal.

### Magnesium Chloride as a Likely Adjuvant to Anesthesia

Although MgCl_2_ has been suggested as an “anesthetic” for *Octopus vulgaris* by several authors (García-Franco, 1992; Pugliese et al., 2016; Butler-Struben et al., 2018), it is a muscle relaxant which renders the preparation immobile but cannot ensure that the animal is pain free and there has been substantial controversy on this issue (Lewbart and Mosley, 2012). In support its action as a neuromuscular blocking agent (NMBA), it is now clear that pre-treatment of isolated rat phrenic nerve-hemidiaphragm preparations with MgCl_2_ prior to blockade with rocuronium reduces the efficacy of potent NMBAs such as sugammadex (Sung et al., 2017), while also decreasing the time to onset of neuromuscular blockade induced by rocuronium (Rotava et al., 2013). It should, however, be noted that magnesium sulfate proved inadequate for procedural sedation when combined with ketamine in a recent randomized clinical trial (Azizkhani et al., 2018). Magnesium salts have been used as an adjunct to clinical anesthesia (Elsersy et al., 2017; Elsonbaty and Isonbaty, 2017), and may attenuate vincristine-induced neuropathic pain (Bujalska et al., 2009) and chronic diabetic neuropathic pain (Rondon et al., 2010) in rat models, where they have also been found to reduce inflammatory pain (Srebro et al., 2018), probably due to the blocking action of Mg^2+^ on NMDA receptors. Such receptors are well known both in cephalopods (Di Cosmo et al., 2004, 2006) and gastropods (Moccia et al., 2009). Thus, it is probable that magnesium chloride acts as a muscle relaxant and as a mild central sedative analgesic (James, 1992; De Oliveira et al., 2013). External application of MgCl_2_ should diminish the early excitatory phase of anesthesia, well known in mammals, humans (Guedel, 1937), in *Lymnaea* (McCrohan et al., 1987) and *Octopus vulgaris* (Polese et al., 2014), but not normally observable in current clinical practice due to the use of muscle relaxants. Thus, MgCl_2_ may be useful as an adjunct to anesthesia.

### Anesthetizing *Octopus vulgaris*

As described above, the gastropod *Lymnaea stagnalis* has proved to be a useful model for the study of anesthetics and the clinical inhalational anesthetics halothane, enflurane and isoflurane have all been used to induce anesthesia in *Lymnaea* (Cruickshank et al., 1985a,b,c; Girdlestone et al., 1989b; Winlow et al., 1995, 1998). In a recent paper the inhalational anesthetic isoflurane was chosen to develop an appropriate protocol for anesthetization of *Octopus vulgaris* (Polese et al., 2014) because it is very stable (Ebert, 2006) reliable, easily available, relatively inexpensive, and “can produce adequate muscle relaxation for any surgical procedure” (Eger, 1981). The first response to applied anesthetic in *Octopus* is the defense posture and the animal becomes hyperexcitable (Polese et al., 2014), but preliminary experiments in Di Cosmo’s Laboratory suggest that MgCl_2_ limits hyperexcitability thereby enhancing the actions of isoflurane.

#### Behavioral Criteria for Anesthesia in *Octopus vulgaris*

**FIGURE 8:**
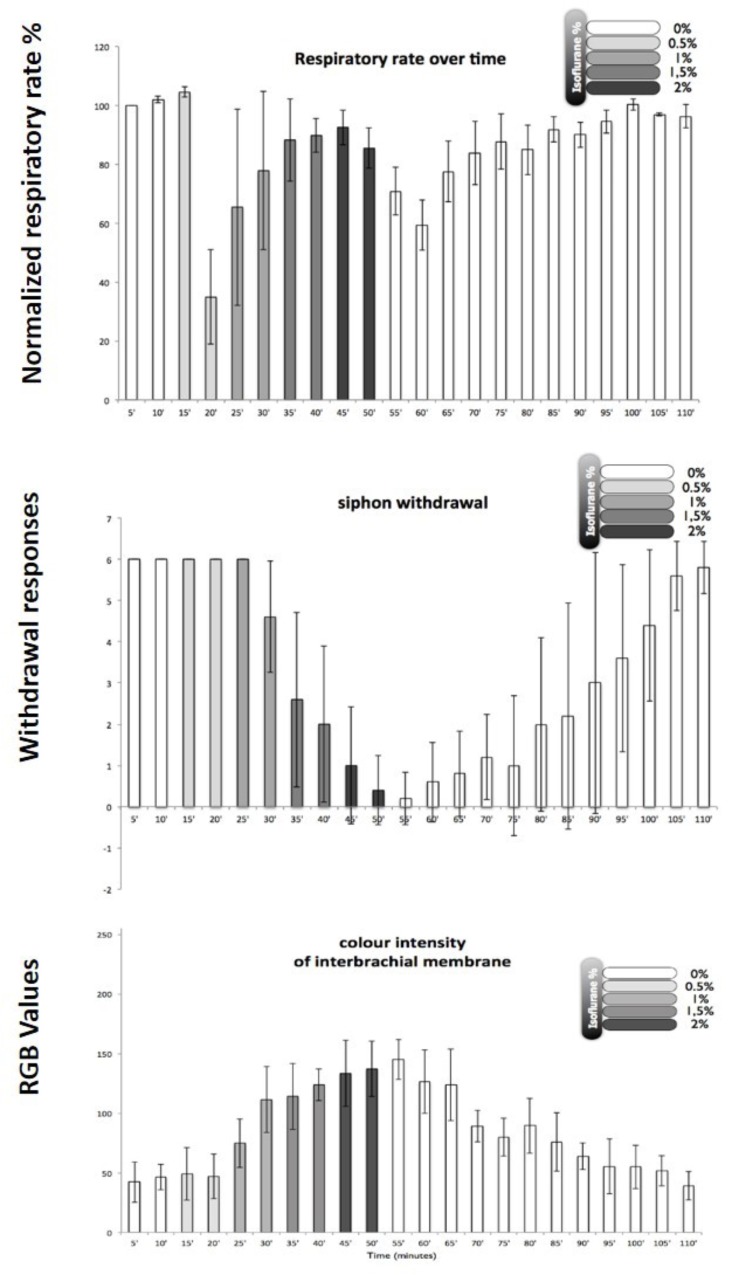
Illustration of behavioral criteria for anesthesia in *Octopus vulgaris*. Effects of isoflurane on respiratory rate **(upper)**, siphon withdrawal **(middle)** and color intensity of the interbrachial membrane **(lower)** in *Octopus vulgaris.* Normalized respiratory rate as determined by the number of mantle contractions in 10 animals at 5 min intervals. The relative strength of siphon withdrawal in response to a touch stimulus (6 = strong, 4 = medium, 2 = low and 0 = none). The color intensity was measured using imageJ software and the RGB model, whereby a zero intensity (value 0) for each component gives the darkest color (no light = black) and full intensity for each component gives white (value 255). Thus the higher recorded value, the paler the interbrachial membrane and *vice versa*. After 50 min the seawater tank was refreshed and the isoflurane flow was switched off. Error bars are standard deviations. (Modified from Polese et al., 2014 – with permission from Journal of Aquatic Animal Health, 2014, Publisher John Wiley and Sons).

In order to develop a method for induction of isoflurane anesthesia of *Octopus vulgaris*, it was necessary to set appropriate practical criteria for observing changes in the animal’s physiology and behavior. The most obvious physiological criterion to observe was the respiratory rate as judged by the frequency of respiratory pumping particularly as most clinical anesthetics depress minute ventilation in mammals (Berge and Warner, 2000). In addition, two behavioral tests were applied: (i) the withdrawal response to stimulation of the arms and siphon because withdrawal responses are used to test for depth of anesthesia in humans and other animals and are diminished in a dose-dependent manner by inhalational anesthetics in the pulmonate mollusc *Lymnaea stagnalis* (Girdlestone et al., 1989b); (ii) color change, which is common in cephalopods and is known to be under central motor control. Using these criteria, it was found that in octopuses the best approach to induction of anesthesia was to slowly increase the isoflurane concentration from 0.5% v/v to 2.0% v/v over a period of about 40 min. Using these criteria, *Octopus* were anesthetized with isoflurane, again in the clinical range. Different animals of the same size responded with similar behavioral changes as the isoflurane concentration was gradually increased (**Figure 8**). After gradual application of 2% isoflurane when all the responses indicated deep anesthesia (i.e., minimal withdrawal responses, pale color, but maintained respiratory rate after an initial reduction – see **Figure 8**)^1^, the animals recovered within 45–60 min in fresh aerated sea water. Based on previous findings in gastropods, we believe that the process of anesthesia induced by isoflurane is similar to that previously observed in *Lymnaea*.

Further studies on anesthetic mechanisms in a range of cephalopods are necessary and clues as to their likely responsiveness to other anesthetic agents might be gained from background studies on other molluscs such as *Lymnaea*, where probable cellular targets for anesthetic actions on behavior can be identified (Rozsa, 2000; Winlow and Polese, 2014). It is likely that the best and most easily controllable anesthetic agents are likely to be volatile anesthetics which have been shown to work well on gastropod molluscs as well as on vertebrates.

#### Inappropriate Anesthetic Agents for *Octopus vulgaris*

Preliminary experiments indicate that neither midazolam nor propofol are suitable agents for anesthetizing *Octopus vulgaris* when bath applied. It may be possible to administer them systemically by injection after use of a muscle relaxant, but this approach is much less controllable than the use of volatile anesthetics which are easily removed from the bathing medium. Nor do we recommend the use of local anesthetics without further detailed study. Midazolam, like other benzodiazepines, is believed to interact with the gamma-aminobutyric acid (GABA)-benzodiazepine receptor complex to prolong its inhibitory actions. It may be used as an anesthetic, particularly in children, and also acts as an amnesic. It may also be used to pre-medicate patients before general anesthesia. However preliminary experiments indicate that midazolam does not anesthetize *Octopus vulgaris*, even at high concentrations, but on the contrary causes a general increase in excitability, including escape behavior, chromatophore flashing and eventually inking. Thus, midazolam is inappropriate as either an anesthetic or for pre-anesthesia of *Octopus*. Propofol immersion is inappropriate because with a gradual increase in propofol concentration over about 20 min, the respiratory rate at first stayed constant, then declined, became very unstable and dropped to zero at which point the animals (*n* = 2) inverted themselves and died.

## Conclusion

Clinical volatile anesthetics are effective on both gastropods and cephalopods as well as vertebrates and are more easily controllable than systemic general anesthetics. However, further work is required to find the optimal way to anesthetize *Octopus vulgaris* and related cephalopods and there is compelling data to indicate that pretreatment with a muscle relaxant followed by a volatile anesthetic will be the best approach. Elucidation of the neuronal circuits underlying the behavioral response to anesthetic agents are also required and, in conjunction with primary neuronal cultures, recently developed from Octopus brain (Maselli et al., 2018), they present us with opportunities to study anesthetic effects at the cellular level in cephalopods for the first time.

## Author’s Note

With apologies to Jane Austen, 1811, for the title.

## Author Contributions

WW wrote the review in consultation with ADC and with critiques from GP, H-FM, and IA.

## Conflict of Interest Statement

The authors declare that the research was conducted in the absence of any commercial or financial relationships that could be construed as a potential conflict of interest.

## Footnotes

1 http://www.sciencemag.org/news/2014/11/video-how-anesthetize-octopus
